# Expanding TOR Complex 2 Signaling: Emerging Regulators and New Connections

**DOI:** 10.3389/fcell.2021.713806

**Published:** 2021-07-30

**Authors:** Peng An, Wenyi Xu, Junjie Luo, Yongting Luo

**Affiliations:** Beijing Advanced Innovation Center for Food Nutrition and Human Health, Key Laboratory of Precision Nutrition and Food Quality, Department of Nutrition and Health, China Agricultural University, Beijing, China

**Keywords:** TORC2, plasma membrane, pathogen, metabolites, feedback, crosstalk

## Abstract

Almost three decades after its seminal discovery, our understanding of the remarkable TOR pathway continues to expand. As a TOR complex, TORC2 lies at the nexus of many signaling pathways and directs a diverse array of fundamental processes such as cell survival, proliferation, and metabolism by integrating environmental and intracellular cues. The dysregulation of TORC2 activity disrupts cellular homeostasis and leads to many pathophysiological conditions. With continued efforts at mapping the signaling landscape, the pace of discovery in TORC2 regulation has been accelerated in recent years. Consequently, emerging evidence has expanded the repertoire of upstream regulators and has revealed unexpected diversity in the modes of TORC2 regulation. Multiple environmental cues and plasma membrane proteins that fine-tune TORC2 activity are unfolding. Furthermore, TORC2 signaling is intricately intertwined with other major signaling pathways. Therefore, feedback and crosstalk regulation also extensively modulate TORC2. In this context, we provide a comprehensive overview of revolutionary concepts regarding emerging regulators of TORC2 and discuss evidence of feedback and crosstalk regulation that shed new light on TORC2 biology.

## Introduction

In eukaryotes, the evolutionarily conserved Ser/Thr kinase target of rapamycin (TOR) functions as a central integrator when growth factors, nutrients, and cellular energy status favor anabolism. These stimuli activate TOR metabolic pathways and ultimately drive cell growth and survival (Saxton and Sabatini, 2017). In mammalian cells, mTOR complex 2 (mTORC2) differs from mTORC1 in many ways, such as accessory components and sensitivity to rapamycin, as well as upstream inputs, downstream effectors, and cellular functions. Besides two shared subunits, mTOR and mLST8, mTORC2 specifically consists of obligate scaffolds Rictor and mSin1 and, unlike mTORC1, is insensitive to acute rapamycin inhibition (Saxton and Sabatini, 2017). For clarity, TORC2 refers to TOR complex 2 in both yeast and a general situation, while mTORC2 refers specifically to mammalian systems in this review.

TORC2 exerts pleiotropic effects on cellular metabolism and homeostasis primarily through activation of downstream effectors. Therefore, TORC2 activity is defined as its ability to phosphorylate downstream targets, which are currently well recognized as AGC kinases, including Akt, PKC, and SGK1 (Zoncu et al., 2011). The most characterized and widely used TORC2 target is Akt-S473 (in a hydrophobic motif of Akt), although this site can also be targeted by other kinases such as ILK and DNA-PK in a specific cellular context (Luo et al., 2018). Akt-S473 is phosphorylated by a canonical posttranslational mechanism, which can be recapitulated in an in vitro kinase assay with immunoprecipitated TORC2. In analogy to Akt, TORC2 also phosphorylates PKC and SGK1 at their corresponding hydrophobic motif residues. One recent study convincingly identified a conserved motif termed TIM (TOR-interaction motif) in the catalytic domain of AKT and PKC as a new TORC2 target (Baffi et al., 2021). In this model, the phosphorylation of TIM acts as the first rate-limiting step that facilitates subsequent PDK1-mediated activation loop phosphorylation and triggers intramolecular hydrophobic motif autophosphorylation to fully activate the kinase. This important finding reveals the long-elusive role of TORC2 in the regulation of AGC kinase. Upon activation by TORC2, these AGC kinases also selectively regulate multiple substrates, such as FoxO1/3a and NDRG1, leading to downstream effects (Manning and Toker, 2017). Although TORC2 activity might specifically initiate a downstream signaling axis in a cell/tissue-dependent manner (discussed below), the rigorous approach to document TORC2 activity requires measurement of the phosphorylation status of all these AGC kinases as well as their substrates. Some studies discussed here indeed take this rigorous approach, and Akt-S473 was most widely used to track the changes of TORC2 activities (Brown et al., 2011; Festuccia et al., 2014; Xu et al., 2019).

For proper maintenance of cellular homeostasis, the activation of TORC2 should be precisely regulated inside the cell. There are many indicators that dysregulation of TORC2 activity underlies a wide spectrum of human pathologies, including cancer, metabolic disorders, autoimmune diseases, aging, and neuronal-related diseases (Tang et al., 2016; Guri et al., 2017; Chen et al., 2019; Cook et al., 2020). Therefore, understanding the regulatory basis for TORC2 activation is becoming important and may hold the key to rethinking many fundamental pathophysiological processes. Meanwhile, knowing how to modulate TORC2 activity presents therapeutic opportunities for the treatment of a broad range of diseases.

Partially due to the lack of acute pharmacological agents that tease apart the two TOR complexes, the regulation of TORC2 is relatively less studied and still poorly defined compared to that of TORC1. However, emerging evidence in recent years has greatly expanded the repertoire of upstream regulators of TORC2. It is becoming clear that, unlike TORC1 activity that mainly depends on lysosomes (Kim and Guan, 2019), the regulation of TORC2 activity occurs at distinct cellular compartments. For example, TORC2 activity is tightly linked to the plasma membrane, where this kinase can be directly and extensively regulated by diverse exogenous cues and membrane-bound proteins. Meanwhile, TORC2 responds to multiple intracellular cues, such as small GTPases, reactive oxygen species (ROS), and ribosomes, and each shows distinct modes for TORC2 regulation. As a typical cellular signaling program, TORC2 activity is also subjected to feedback regulation from multiple downstream nodes that spatiotemporally terminate or boost TORC2 signaling. In addition, TORC2 signaling presents extensive crosstalk with other major signaling pathways, such as Hippo, WNT, and Notch pathways. These highly interconnected and coordinated networks significantly influence TORC2 activity and signaling relay, and greatly broaden its range of biological activities.

In this review, we focus on current major developments in TORC2 regulation that illustrate how the expanding spectrum of emerging inputs influences its activity. We also discuss how TORC2 signaling is rewired and intersects with other major pathways through feedback and crosstalk regulation, which also profoundly modulate TORC2 signaling.

## Diversified Regulation of TORC2 Activity

### Regulation Through the Plasma Membrane

**FIGURE 1:**
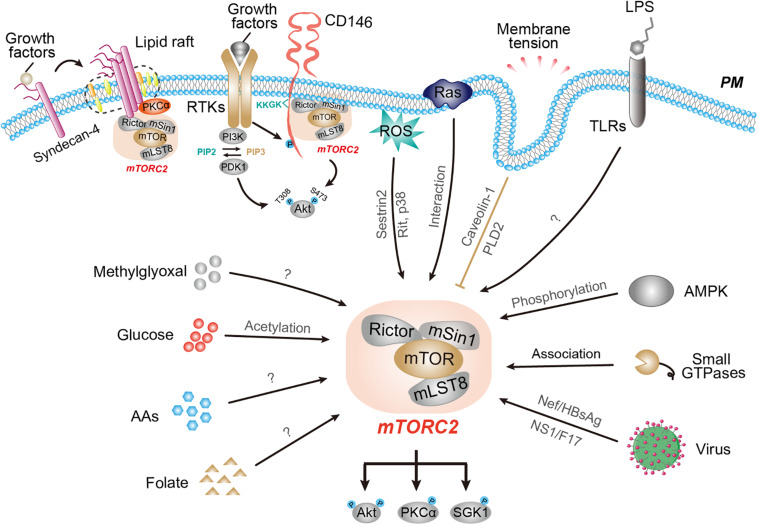
The responses of mTORC2 activity to various environmental and intracellular cues. Schematic representation of mTORC2 activity in response to a wide array of upstream regulators, including signals from the plasma membrane, metabolites, and pathogens as well as intracellular cues. Interference with membrane tension activates mTORC2 through caveolin-1 and PLD2. RTK signaling activates mTORC2 via PI3K-dependent and -independent mechanisms. Oncogenic Ras and CD146 associate with and activate mTORC2 at the plasma membrane. The activity of mTORC2 is regulated by nutrients, such as glucose, amino acids, and folate. Several intracellular cues, such as AMPK, small GTPases, ribosomes, and ROS also regulate mTORC2 activity through distinct mechanisms.

TORC2 activity is tightly associated with the plasma membrane. Although the precise subcellular localization of TORC2 is still under debate, at least some TORC2 in both mammalian cells and yeast appears to be on the plasma membrane as detected by microscopy and biochemical fractionation approaches (Partovian et al., 2008; Gao et al., 2011; Berchtold et al., 2012). Therefore, signals generated at the plasma membrane, such as receptor tyrosine kinase (RTK), oncogenic Ras, cell adhesion receptors, membrane tension, as well as bacteria-derived pathogens, effectively modulate TORC2 activity through distinct mechanisms (Figure 1).

#### RTK Signaling

Growth factor signaling through RTK and PI3K pathways has been well recognized as the major activating signal for TORC2. In mammalian cells, the mTORC2-dependent mTOR autophosphorylation at Ser2481, a marker for intact mTORC2 (Copp et al., 2009), and *in vitro* mTORC2 activity, using Akt-S473 as a substrate, are stimulated by growth factors (Frias et al., 2006; Yang et al., 2006). Furthermore, mTORC2 appears to phosphorylate SGK1 in response to growth factors even though SGK1 lacks a pleckstrin homology (PH) domain and is activated independently of membrane recruitment (Garcia-Martinez and Alessi, 2008).

However, the regulatory mechanism governing growth factor-induced mTORC2 activation is still under debate. One possible mechanism might occur via mTORC2 recruitment to the plasma membrane or specialized membranous structures where mTORC2 could be modulated by RTK signaling with high efficiency. One proposed PI3K-dependent model is that, upon PI3K activation by RTKs, PIP3 interacts with mSin1-PH to unmask the inhibition of mSin1 on the mTOR kinase domain, while recruiting mTORC2 to the plasma membrane where recruited Akt (via Akt-PH domain) gets phosphorylated (Liu et al., 2015). In this study, only Akt-S473 was used in assays for measurement of mTORC2 activity. However, it should be noted that Akt-S473 phosphorylation by mTORC2 inside a cell requires PIP3, which accumulates in response to PI3K stimulation by many growth factor receptors (Manning and Toker, 2017). An alternative interpretation of growth factor-induced Akt-S473 phosphorylation is PIP3-dependent recruitment of Akt to constitutively active membrane-associated mTORC2. Whether and how PI3K is involved in growth factor-targeted mTORC2 activity is not clear.

Recent biochemical labeling and imaging studies using a compartment-specific mTORC2 activity reporter, dubbed LocaTOR2, revealed an alternative mechanism for mTORC2 activation. In mammalian cells, the activity and localization of mTORC2 via mSin1-PH at the plasma membrane is PI3K- and growth factor-independent, and membrane recruitment of Akt is sufficient for mTORC2-mediated Akt-S473 phosphorylation in response to growth factors (Ebner et al., 2017). This is supported by the observation that myristoylated Akt at the plasma membrane results in its hyperphosphorylation (Andjelkovic et al., 1997) and yeast TORC2 localizes to the plasma membrane and membrane-proximal vesicles where it promotes cell survival (Berchtold and Walther, 2009). Nevertheless, mTORC2 activity in the endosomal pool responds to PI3K (Ebner et al., 2017), suggesting the existence of spatially separated mTORC2 populations with distinct sensitivity to growth factors. It is therefore still unknown whether and to which extent growth factors can directly modulate mTORC2 activity. Meanwhile, how does mTORC2 at the plasma membrane stay constitutively active? One possible explanation is that other membrane proteins or extracellular cues might also account for mTORC2 membrane anchoring and activity (see discussion below).

#### Oncogenic Ras

The majority of early studies suggest that mTOR is not regulated by Ras through direct contact but, rather, distally via Ras stimulation of PI3K and mitogen-activated protein kinase (MAPK) pathways (Thorpe et al., 2015; Kim et al., 2017). However, recent work combining biotin labeling of proteins (BioID) proteomics with CRISPR screening identified mTORC2 as a functionally direct effector of oncogenic Ras mutants in human cancer cells (Kovalski et al., 2019). It was found that active Ras directly and selectively interacted with mTORC2 by binding the Ras-binding-domain (RBD) of mSin1 and mTOR kinase domain via the Ras effector interaction domain. Therefore, oncogenic Ras increased mTORC2 kinase activity, as indicated by LocaTOR2, in cells at the plasma membrane but not at other cellular sites to positively regulate cell proliferation. Blocking mTORC2 localization and association with Ras at the plasma membrane by deleting the RBD and PH domains of mSin1 impairs mTORC2 enzymatic activity and Ras-dependent tumorigenesis (Kovalski et al., 2019). Besides being a downstream effector of Ras, mTORC2 might also feedback to Ras signaling. For example, mSin1 expression inhibited the activation of ERK and JNK signaling pathways by Ras, suggesting that mSin1 was a mammalian Ras inhibitor and mTORC2 may contribute to negative feedback regulation of Ras activity (Schroder et al., 2007).

#### Cell Adhesion Receptors

Our recent study identified an essential cell adhesion receptor CD146 that directly linked mTORC2 activation with extracellular growth factors to enable cell proliferation and survival (Xu et al., 2019). The activation of RTKs phosphorylates the only tyrosine residue, Y641, in the cytoplasmic tail of CD146, which enables the juxtamembrane positively charged KKGK motif to associate with Rictor. Through this interaction, CD146 activates mTORC2 (as evidenced by increased phosphorylation of Akt, PKCα/β, SGK1, NDRG1, and Foxo1/3) by protecting Rictor from ubiquitin proteasome-mediated degradation, thereby promoting cell proliferation. Mutation of Y641 or deletion of KKGK disrupts the ability of CD146 to bind Rictor and activate mTORC2 in response to multiple growth factors, including VEGF, bFGF, insulin, and IGF-1, indicating that CD146 directly links divergent environmental growth cues with mTORC2 activity. It is interesting to note that the regulation of mTORC2 activity by CD146 showed no effect on PI3K and mTORC1 activity, because the CD146-KKGK motif specifically targets the mTORC2 unique subunit Rictor. This observation represents a generic mechanism for mTORC2-selective regulation and a strategy for specific inhibition of mTORC2 activity.

The observation that mTORC2 activation depends on its association with juxtamembrane positively charged amino acid cluster of CD146 might represent a general mechanism for the regulation of mTORC2 activity at the plasma membrane. It should be noted that several Ras proteins also contains a similar polybasic motif in their juxtamembrane hypervariable region (Simanshu et al., 2017), which might form a similar structural basis for binding and subsequent activation of mTORC2 by Ras. This was also supported by the finding that the reporter LocaTOR2 system contains a similar positively charged motif (Ebner et al., 2017) and Rictor downregulation drastically reduced the interaction between Ras and mTORC2 components (Kovalski et al., 2019). Meanwhile, a similar motif is also present in the juxtamembrane portion of several other cell adhesion molecules, such as L-selectin (Ivetic et al., 2002, 2004), ICAM-1/2, CD43, CD44, and L1CAM (Yonemura et al., 1998; Cheng et al., 2005; Oh et al., 2007). It will be interesting to see in the near future whether these juxtamembrane motifs within these integral membrane proteins can also serve as a default signal for Rictor binding and mTORC2-specific regulation.

#### Syndecan-4

In endothelial cells, mTORC2 was observed localized to plasma membrane raft domains. Syndecan-4, which is a single-pass transmembrane proteoglycan, recruits PKCα to the membrane raft to regulate PKCα activity; this, in turn, is required for appropriate mTORC2 localization to rafts and subsequent phosphorylation of Akt, PKC, GSK-3, eNOS, and Foxo1/3a in response to FGF2 (Partovian et al., 2008). This proposed mechanism for mTORC2 activation supports the concept of rafts as dynamic signal transduction platforms whose transient recruitment and stabilization of signaling complexes within rafts produces a bulk and efficient cellular signaling response (Lajoie et al., 2009). Nevertheless, the mechanism by which PKCα regulates mTORC2 recruitment to rafts is unknown, and discovery efforts are complicated by the fact that PKCα is known to be a downstream target of mTORC2.

#### Membrane Tension

Membrane tension is generated as a response to any application to the membrane surface of external forces (Kozlov and Chernomordik, 2015). Localization to the plasma membrane supports a downstream role of TORC2 in membrane tension. The observation that membrane tension could regulate TORC2 activity originally comes from yeast. In yeast, interference with plasma membrane tension activates TORC2 (Berchtold et al., 2012). In this step, the PH domain-containing plasma membrane protein Slm1/2 acts as an upstream regulator of TORC2 activation. Upon plasma membrane stress that reduces membrane tension, such as inhibition of sphingolipid biosynthesis or mechanical stretching of the plasma membrane, Slm proteins are partitioned away from membrane compartments called eisosomes and relocalized to the specialized membrane domain MCT (membrane compartment containing TORC2), where Slm1/2 associates with and activate TORC2. Slm1/2 also facilitates recruitment of the AGC kinase Ypk1 to proximity of TORC2 for phosphorylation and activation. Activated Ypk1 in turn promotes biosynthesis of sphingolipids, which leads to the resolution of plasma membrane stress and ultimately TORC2 deactivation (Berchtold et al., 2012; Niles and Powers, 2012). Therefore, membrane tension and TORC2 activity regulate each other through a feedback mechanism.

The regulation of TORC2 activity by membrane tension is conserved in mammalian cells. Vertebrate-specific membrane invaginations called caveolae has been suggested to function analogously to eisosomes. Mechanical stretch, which leads to rapid disassembly of caveolae, triggers mTORC2-dependent phosphorylation of Akt-S473 (Kippenberger et al., 2005; Sinha et al., 2011). This process requires caveolin-1, the defining protein of caveolae, in epithelial cells and vascular smooth muscle cells (Sedding et al., 2005; Zhang et al., 2007). Similarly, TORC2 activity (as indicated by Akt phosphorylation) is increased following hypo-osmotic shock-induced stretching of the plasma membrane in both mammals and yeast (Chen et al., 2011; Muir et al., 2014). During neutrophil chemotaxis, acute stretching of the plasma membrane leads to an increase in mTORC2 activity mediated by phospholipase D2 (PLD2) to limit actin nucleation, leading to cell polarity and migration (Diz-Munoz et al., 2016). Collectively, it can be concluded that TORC2 activity responds to membrane tension in both mammals and yeast. Since most of these studies have been limited to cell models, the pathophysiological consequences of TORC2 regulation by membrane tension remain to be determined.

#### Bacteria-Derived Pathogens

The engagement of Toll-like receptors (TLRs) by their cognitive ligands [such as lipopolysaccharide (LPS)] acutely activates mTORC2 as indicated by the phosphorylation of Akt, PKCα/β, SGK1, NDRG1, and Foxo1/3, and mTORC2 negatively feedbacks to TLR signaling to suppress the pro-inflammatory response in multiple types of mammalian cells (Brown et al., 2011; Festuccia et al., 2014; Sato et al., 2018). However, the mechanism regulating mTORC2 activation by TLRs remains unclear. In macrophages and epithelial cells, LPS-induced mTORC2 activity could be modulated by other bacteria-derived pathogens. For example, the recognition of β-barrel outer membrane proteins (OMPs) of Gram-negative bacteria by the cell surface receptor SlamF8 could reduce the phosphorylation of mTOR at S2481 to inhibit mTORC2 and Akt activation in response to LPS (Chaudhary et al., 2018). Therefore, OMPs counteract LPS-induced mTORC2 activity to inhibit intracellular bacterial replication through unknown mechanisms.

### Regulation Through Metabolites

TORC1 activity is mainly dependent upon sensing amino acids and tightly regulated by other nutrient inputs to support cell metabolism and growth throughout evolution; TORC2 activity is not as tightly coupled to nutrient availability. In recent years, emerging evidence has suggested that TORC2 activity also responds to and can be modulated by distinct nutrients and metabolites. However, the robustness and pathophysiological implications will require further validation.

#### Glucose

TORC2 activity is regulated by glucose. In human glioblastoma, for example, extracellular high glucose conditions activate mTORC2 (as indicated by Akt-S473 and NDRG1-T346) via acetyl-CoA-dependent acetylation of Rictor. Rictor acetylation is maintained to form an autoactivation loop of mTORC2 even in the absence of growth factor receptor signaling (Masui et al., 2015), nominating mTORC2 as a central node for integrating growth factor signaling with nutrient availability. p300, a histone acetyltransferase, and SIRT1 modulate the acetylation of Rictor, which is critical for insulin-stimulated Akt activation (Glidden et al., 2012). However, whether mTORC2 activity responds to glucose in other types of cells and the physiological consequence remain unclear.

#### Amino Acids

Amino acids can both activate and inhibit TORC2 activity depending on the cellular context. For example, amino acids positively regulate mTORC2 activity (as indicated by increased phosphorylation of Akt-S473 and GSK-3), together with mTORC1, via class I PI3K and only mTORC2 is required for purine synthesis in multiple human cells (Tato et al., 2011; Saha et al., 2014). On the other hand, amino acids can also exert inhibitory effects on mTORC2. For example, amino acids can release the restraint of T cell proliferation by mTORC2, and Rictor-deficient T cells continue to proliferate despite the inadequate amino acids (Van de Velde and Murray, 2016; Van de Velde et al., 2017). It is possible that amino acids activate the cell cycle by inhibiting mTORC2. However, the reason for the opposite role of amino acids in mTORC2 activity and how amino acids regulate mTORC2 remain largely unknown.

#### Folate

TORC2 activity (together with TORC1) responds to folate stimulation without a clear mechanism (Silva et al., 2017). Folate deficiency in pregnant mice and trophoblasts caused a marked inhibition of mTORC2 signaling (as evidenced by decreased phosphorylation of Akt-Ser473, PKC-Ser657, and SGK-Ser422) and decreased activity of key amino acid transporters, resulting in restricted cell growth (Rosario et al., 2017a). In turn, mTORC2 signaling is critically involved in folate availability and cell growth and proliferation. It has been shown that folate sensing and uptake in trophoblasts involves both mTOR complexes and requires the proton coupled folate transporter (PCFT; solute carrier 46A1) (Rosario et al., 2017b). Although the mechanism remains unexplored, the responsiveness of both mTOR complexes to folate might explain the linkage between low maternal folate and restricted fetal growth.

#### Methylglyoxal

Methylglyoxal provides another example that potentially links cellular metabolic status with TORC2 activity with unknown mechanisms. As a typical reactive intermediate derived from glycolysis or gut microbes, methylglyoxal stimulates mTORC2 activity, which is responsible for the phosphorylation of Akt-S473 and GSK3β-S9 in human colorectal cancer cells (Bellier et al., 2020). The activation of this kinase by methylglyoxal is also conserved in lower organisms with more diversified downstream outputs. In *Saccharomyces cerevisiae*, methylglyoxal activates TORC2-PKC as well as TORC2-Akt signaling (Nomura and Inoue, 2015), while methylglyoxal promotes longevity by activating the TORC2-SGK-1/DAF-16 signaling axis in *C*. *elegans* (Shin et al., 2020). Preferential activation of the TORC2 signaling axis by methylglyoxal is currently unclear. One possibility is that methylglyoxal might activate distinct TORC2 pools that localize to specific cellular compartments or mediate the recruitment of distinct effectors to TORC2 in different cellular contexts. The common physiological significance of TORC2 activation by methylglyoxal also remains unknown.

### Regulation Through Virus

The evidence that TORC2 activity responds to viruses or viral proteins is mainly from studies of mammalian cells. Viruses employ elaborate strategies for their replication and survival during infection by regulating mTORC2 through distinct mechanisms. In macrophages, HIV-1 infection initiates an interaction of POTEE with the viral protein Nef to activate mTORC2, leading to dissemination of macrophages in other systems (Vekariya et al., 2018). In hepatocytes, a high level of HBV protein HBsAg deactivates mTORC2 by increasing endoplasmic reticulum stress, leading to enhancement of Fas-mediated apoptosis (Jing et al., 2018). In neuronal cells, Zika virus (ZIKV) infection activates mTORC2 through unknown mechanisms. The activation of mTORC2 facilitates ZIKV replication by negatively regulating autophagy (Sahoo et al., 2020). Upon influenza virus infection, the viral protein NS1 promotes mTORC2-mediated Akt-S473 phosphorylation, which inhibits cell apoptosis (Kuss-Duerkop et al., 2017). Poxvirus protein F17 binds and sequesters both Rictor and Raptor to disrupt the mTORC1-mTORC2 regulatory circuit, thereby blocking the STING-IFNγ antiviral response without affecting mTOR-mediated viral protein synthesis (Meade et al., 2018). It should be noted that only Akt-S473 was used for documenting mTORC2 activity in most of these studies. Therefore, it is still unclear how and to what extent viruses modulate mTORC2 activity. Collectively, the regulation of mTORC2 by viruses would form a core strategy during evolution to facilitate viral survival in host cells, simultaneously dampening host cytosolic sensing and immune responsiveness.

### Regulation Through Intracellular Cues

#### AMPK

AMP-activated protein kinase (AMPK) is an energetic stress kinase that promotes catabolic and suppresses anabolic metabolism coordinately to restore energy balance (Lin and Hardie, 2018). Given such intimate regulation of metabolism by AMPK, it may play a role in TORC2 regulation. Indeed, various AMPK activators promote phosphorylation of Akt-S473 in an AMPK-dependent manner in cultured mammalian cells (Kazyken et al., 2019). This AMPK-mediated activation of mTORC2 occurs independently of mTORC1-mediated negative feedback. AMPK associates with mTORC2 and directly phosphorylates mTOR and possibly Rictor to increase mTORC2 activity. Thus, in addition to the role of AMPK in inhibiting mTORC1, AMPK also activates mTORC2 by direct phosphorylation of mTORC2 components during energetic stress to enhance cell survival (Lin and Hardie, 2018; Kazyken et al., 2019). Therefore, the emerging concept is that mTORC2 is the convergent point for anabolic (e.g., insulin/PI3K) and catabolic (e.g., AMPK) signals to coordinate cellular metabolic homeostasis. It will be important to identify where in the cell AMPK activates mTORC2, and specific AMPK-mediated regulatory sites in mTORC2 components that reflect its activity, localization, and/or substrate specificity.

#### Small GTPases

GTPases play essential roles in regulating TORC2 activity. Rictor and mSin1 contain RasGEFN (N-terminal to Ras guanine nucleotide exchange factor) and Ras binding domain, suggesting a correlation with GTPase (Gaubitz et al., 2016). Indeed, Rit, a Ras-family GTPase, activates mTORC2 (Akt-S473 phosphorylation) in response to ROS by binding mSin1 (Cai and Andres, 2014). GTPases in both GTP- and GDP-bound forms regulate TORC2 activity by physically interacting with TORC2. For example, the GTP-bound form of Rhy1, a Rab-family GTPase, is shown to bind and activate yeast TORC2 (Tatebe et al., 2010; Hatano et al., 2015). GTPases also regulate TORC2 activation in GDP-bound forms. Rac1, a Rho-family GTPase, in its GDP-bound form activates mTORC2 (Akt-S473 phosphorylation) in response to growth factors by interacting with mTOR (Saci et al., 2011). Moreover, Rho-GDP promotes Akt phosphorylation by assembling a supercomplex with Ras-GTP and mTORC2. This supercomplex formation is controlled by chemoattractant-induced phosphorylation of Rho-GDP at S192 by GSK-3 (Senoo et al., 2019). The requirement of Rho-GDP and Ras-GTP dimer for direct mTORC2 activation is distinct from that of mTORC1, which depends on RagA/B-GTP and RagC/D-GDP dimers for lysosome localization and Rheb-GTP for subsequent stimulation of mTORC1 enzymatic activity (Saxton and Sabatini, 2017). These studies demonstrate a critical role of GTPases as an upstream component of TORC2 signaling, and challenge the prevailing view that GTPases are downstream effectors of TORC2 that control remodeling of the actin cytoskeleton.

#### ROS

ROS functions as an TORC2 activating factor in protecting cells from oxidative stress from yeast to mammalian cells. In yeast, ROS can activate TORC2 through an unknown mechanism and in turn, TORC2 regulates ROS metabolism to control cell growth and survival (Niles and Powers, 2014; Niles et al., 2014). The activation of mTORC2 activity by ROS is also conserved in mammalian cells. For example, perturbation of mitochondrial energy metabolism due to deficiency of DNA polymerase gamma (Polγ) causes an increase in mitochondrial ROS, which elevates Rictor expression to initiate Rictor-dependent pro-survival autophagy (Dhar et al., 2018). Different mechanisms have been documented for ROS-induced TORC2 activity. In lung cancer, glutamine deprivation stimulates the formation of ROS, and subsequently upregulates expression of Sestrin2, which activates mTORC2 activity (Akt-S473 phosphorylation) and enables cancer cell survival under glutamine depletion (Byun et al., 2017). The ability of ROS to activate mTORC2 is also mediated by Rit and p38 MAPK depending on the cellular context (Cai and Andres, 2014).

### Regulation Through Feedback Mechanism

**FIGURE 2:**
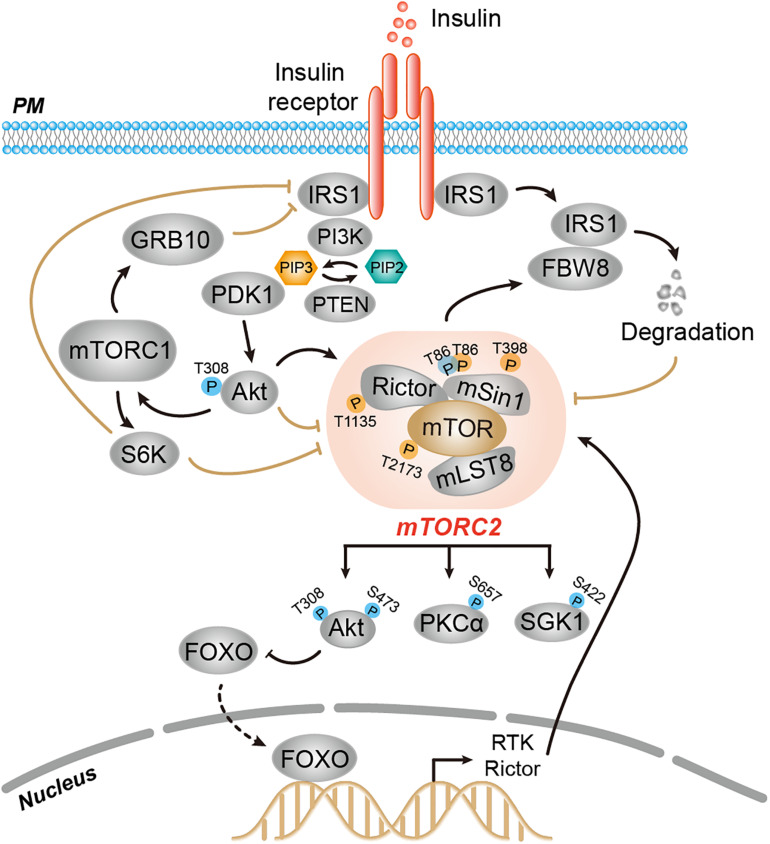
Feedback regulation of mTORC2 activity. Several downstream signals of mTORC2, such as FBW8-IRS, mTORC1-GRB10-IRS, mTORC1-S6K-IRS, Akt, and FoxO, function as negative or positive regulators that impact mTORC2 signaling relay. Upon insulin stimulation, mTORC2 phosphorylates FBW8 to allow the translocation of FBW8 to the cytosol, which mediates ubiquitylation and proteasomal degradation of IRS1, thereby preventing chronic insulin signaling and mTORC2 activation. Insulin-induced mTORC1 phosphorylates and activates Grb10 to inhibit mTORC2 and Akt downstream. The mTORC1 effector S6K1 promotes phosphorylation-dependent degradation of IRS or phosphorylation of Rictor-T1135 and mSin1-T86/398 to dampen mTORC2 signaling. Akt promotes mTOR-T2173 phosphorylation to impair mTORC2 activity. Akt also positively regulates mTORC2 activity by phosphorylating mSin1-T86. Prolonged inhibition of Akt promotes FOXO-dependent transcription of RTK and Rictor.

A hallmark of signaling networks is the presence of multiple nodes with feedback loops. The TORC2 signaling network provides many typical examples of feedback control, a common feature of cellular signaling systems. Similar to other signaling pathways, TORC2 signaling is subjected to negative feedback regulation, which ensures switch-like behavior from initiation of stimulatory signals to termination of the signaling relay in a spatially and timely controlled manner. Therefore, several downstream signals of TORC2 function as negative regulators as TORC2 signaling. Besides negative feedback regulation, TORC2 is also regulated by positive feedback, which acts as a booster for TORC2 signaling (Figure 2).

#### Feedback From TORC2-IRS

Feedback of TORC2 signaling is regulated at the level of TORC2-IRS (insulin receptor substrate). TORC2 responds to insulin signaling through IRS-mediated PI3K activation (Sun et al., 1991; Yang et al., 2006). TORC2 can negatively feedback to IRS1 to decrease insulin receptor signaling (Kim et al., 2012). In fibroblasts, phosphorylation of the F-box protein FBW8 (an E3 ligase component) at Ser86 by mTORC2 allows the translocation of FBW8 to the cytosol upon insulin stimulation. Cytosolic FBW8 mediates ubiquitylation and proteasomal degradation of cytosolic IRS1, thereby preventing chronic activation of insulin signaling and mTORC2 activation (Kim et al., 2012).

#### Feedback From Grb10/S6K

Among mTORC2 downstream effectors, mTORC1 functions as a negative feedback regulator that acutely inhibits mTORC2 signaling through several distinct mechanisms in mammalian cells. First, short-term treatment with rapamycin enhances the activity of Akt in response to insulin and IGF1, suggesting mTORC1 inhibits mTORC2 signaling via feedback regulation between mTORC1 and insulin/PI3K signaling (Manning, 2004). Indeed, insulin can activate both mTORC1 and mTORC2. Activated mTORC1, however, phosphorylates and activates Grb10, a negative regulator of insulin/IGF-1 receptor signaling and thus inhibits downstream mTORC2 and Akt. Second, mTORC1 effector S6K1 promotes phosphorylation-dependent degradation of IRS-1/2, thereby dampening mTORC2 signaling (Hsu et al., 2011; Yu et al., 2011). On the other hand, S6K1 mediates phosphorylation of mTORC2 components Rictor (T1135) and mSin1 (T86/398), which directly decreases mTORC2-dependent phosphorylation of Akt-S473 (Dibble et al., 2009; Julien et al., 2010; Liu et al., 2013).

#### Feedback From Akt

Akt exerts both positive and negative feedback regulation of mTORC2 signaling depending on distinct phosphorylation of mTORC2 components. In human cells, the mTOR protein is phosphorylated at T2173 of the ATP-binding site in the kinase domain in an Akt-dependent manner, which impairs mTORC2 activity; this functions as a negative feedback to directly control mTORC2 signaling (Halova et al., 2013). In contrast, Akt also positively feedbacks to mTORC2, thereby directly potentiating mTORC2 signaling (Yang et al., 2015). The first step is the phosphorylation and activation of Akt-T308 by PDK1, which is followed by an increase in mTORC2 activity upon Akt phosphorylation of mSin1-T86. These subsequent events ensure the phosphorylation of Akt-S473 by mTORC2, thereby establishing positive feedback regulation of mTORC2 signaling (Yang et al., 2015).

#### Feedback From FOXO

The role of FOXO in the feedback regulation of TORC2 signaling comes from cancer studies. In some cancer cells, prolonged inhibition of Akt signaling relieves feedback suppression of RTK expression and activity through FOXO-dependent transcriptional induction, thereby enhancing PI3K-Akt signaling (Chandarlapaty et al., 2011). On the other hand, pharmaceutical inhibition of the PI3K-Akt pathway promotes FOXO-dependent upregulation of Rictor expression and thereby directly feedbacks to mTORC2 activity (Lin et al., 2014).

### Regulation Through Crosstalk With Other Signaling Pathways

**FIGURE 3:**
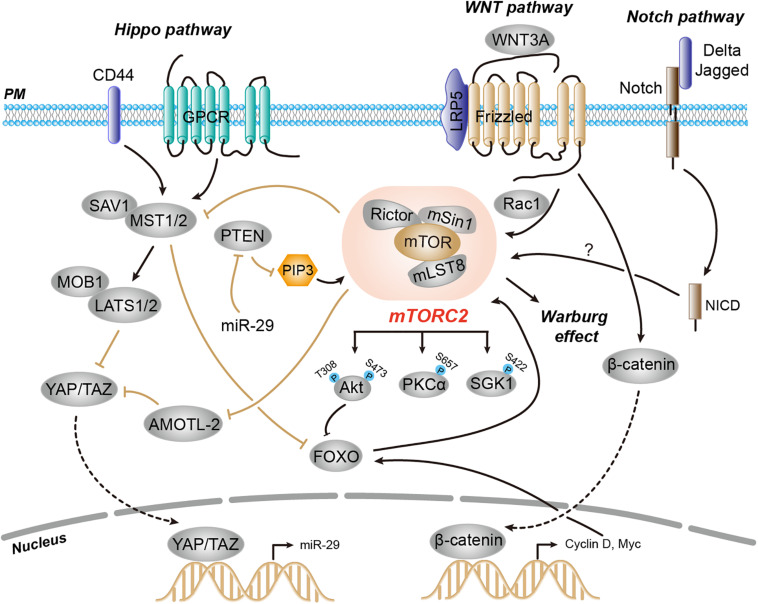
Regulation of mTORC2 activity through crosstalk with other signaling pathways, including Hippo, WNT, and Notch, which act both upstream and downstream of mTORC2. During Hippo inactivation, active and hypophosphorylated YAP translocates to the nucleus and promotes expression of miR-29. The miR-29 targets PTEN mRNA and inhibits its translation, which leads to increased levels of PIP_3_ and activation of both mTORC1 and mTORC2. Another key component of the Hippo pathway, Mst1, phosphorylates and inactivates FOXO1 and substrates of mTORC2-Akt, and thereby regulates mTORC2 downstream signaling. mTORC2 is activated by WNT in a manner dependent on the small GTPase RAC1. Notch signaling promotes mTORC2 activation possibly through PINK1 and mitochondria.

In addition to the independent signaling program of TORC2 that provides compensatory mechanisms, the TORC2 signaling network is extensively intertwined and has several points of crosstalk with other major signaling pathways. These signaling pathways, which include Hippo, WNT, and Notch, act both upstream and downstream of TORC2 pathways (Figure 3; Tumaneng et al., 2012; Esen et al., 2013; Lee et al., 2013; Shimobayashi and Hall, 2014).

#### Hippo Pathway

There is a particularly intimate relationship between the TORC2 and Hippo tumor-suppressor pathways, which engage in bilateral cross-regulation at multiple levels. The Hippo pathway determines precise control of cell size by inhibiting phosphorylation of the transcription co-activator Yes-associated protein (YAP). Upon Hippo inactivation, the active and hypophosphorylated YAP translocates into the nucleus to trigger the transcription of genes encoding cell proliferation (Zhao et al., 2011). It has been demonstrated that YAP upregulates the microRNA (miR) miR-29, which in turn inhibits the expression of PTEN in breast cancer cells. YAP-mediated PTEN downregulation leads to the accumulation of PIP3, which potentiates PI3K signaling and thereby positively activates both mTORC1 and mTORC2 (Tumaneng et al., 2012). On the other hand, mTORC2 also exhibits feedback control to the Hippo pathway via AMOTL2-mediated inactivation of YAP. The mTORC2 phosphorylates AMOTL2 and blocks its ability to bind and repress YAP, leading to glioblastoma cell growth and survival (Artinian et al., 2015). Therefore, the mutual crosstalk between Hippo and mTORC2 tightly controls the balance between cell growth and proliferation.

The bilateral cross-regulation of Hippo and mTORC2 is also mediated by Mst1 kinase, another key component of the Hippo pathway. Mst1 has been shown to phosphorylate and inactivate FOXO1, substrates of mTORC2-Akt, and thereby regulates mTORC2 downstream signaling and cell death in neuronal cells (Lehtinen et al., 2006). In cardiac cells, however, mTORC2 phosphorylates Mst1 kinase and functions as a direct negative regulator of Mst1 activity (Sciarretta et al., 2015).

#### WNT Pathway

Another major signaling pathway that exhibits cross-regulation with TORC2 is the WNT pathway. During osteoblast differentiation, the non-canonical ligand WNT3A activates mTORC2 and therefore induces aerobic glycolysis known as the Warburg effect. This mTORC2-mediated glucose metabolism requires the binding of WNT3A with its co-receptor low-density lipoprotein receptor-related protein 5 (LRP5) but not canonical WNT signaling. Moreover, WNT3A-mediated mTORC2 activation requires RAC1 (Esen et al., 2013). The mTORC2 signaling has another point of cross-regulation with WNT signaling. Activation of the WNT pathway upregulates cyclin D and c-Myc in cancer cells, which eventually activates FOXO to enhance Rictor levels and therefore mTORC2 activation (Chen et al., 2010).

#### Notch Pathway

The Notch pathway is another example of crosstalk with TORC2 signaling. Notch plays critical roles in the control of proliferation and cell fate decisions (Bray, 2006). Notch receptor engagement by cell surface-tethered ligands (Delta and Jagged) on neighboring cells initiates cleavage of the receptor to release the Notch intracellular domain (NICD), which acts as a transcriptional coactivator that promotes gene expression. In multiple cell types including neuroblasts, thymocytes, and leukemia, Notch signaling promotes mTORC2 activation and Akt-S473 phosphorylation, leading to cell survival and an anti-apoptotic response (Perumalsamy et al., 2009; Lee et al., 2012, 2013). Although PTEN-induced kinase 1 (PINK1) and mitochondria have been proposed (Lee et al., 2013), the detailed mechanism for Notch in activation of mTORC2 signaling remains largely unexplored.

## Conclusion and Perspectives

Exciting progress has been made over the last few years in elucidating the complex network of TORC2 signaling and its broad implications in human physiology and pathology. Basic investigations into the genetics, cellular biology, biochemistry, and structural biology of TORC2 have yielded a sophisticated view of how the activity of this multi-protein kinase is regulated. As discussed above, TORC2 acts as a central signaling node that is precisely and exquisitely regulated by a variety of upstream inputs to exert widespread control over many biological processes and maintenance of cellular homeostasis. A natural question is why TORC2 has evolved such diversified modes of regulations. To this end, it is helpful to first consider the biological functions of TORC2, as it centrally controls many fundamental requirements and processes for life, such as cell proliferation, survival, and metabolism. In principle, any perturbation or interference with these processes might converge on this kinase and profoundly impact its activity. Another possible explanation might lie in the molecular composition and structural features of TORC2. TORC2 is a multi-protein kinase consisting of multiple accessory subunits, such as Rictor and Sin1, in addition to the catalytic subunit TOR. These components are large and possess a number of regulatory domains, interactive interfaces, and modifiable sites. These unique compositional and structural features allow TORC2 to respond to a wide array of inputs, and at the same time, permit interactions and post translational modifications that fine-tune TORC2 integrity, assembly, or, perhaps substrate recruitment. Thirdly, like many other protein kinases, the activity of TORC2 heavily relies on its subcellular localization, especially to membranous structures. Therefore, signals that guide TORC2 distribution to specific cellular compartments could also extensively modulate its activity, which permits the sensing of TORC2 to diverse inputs or allow access to distinct substrates at the right time and at the right place. Last but not least, TORC2 signaling has multiple connecting nodes that allow both compensatory feedback control from within TOR signaling, as well as crosstalk with other signaling pathways. These new connections have significantly complicated the regulation of TORC2 activity and its possible biological impacts.

The identification of many emerging upstream inputs and feedback as well as crosstalk mechanisms is revealing the broad scope of TORC2 regulatory modes and is also raising interesting and challenging questions about the regulation of this complex. Signals generated at the plasma membrane are emerging regulators for TORC2 signaling. However, the regulatory mechanisms governing TORC2 activation at the plasma membrane are still incompletely understood. For example, are growth factors and PI3K signaling indeed required for TORC2 activation, and to which extent? How exactly do they control TORC2 membrane targeting and activation? How does membrane tension act on the plasma membrane to regulate TORC2 activation in mammalian cells? Does it involve the dynamics of the cytoskeleton? Could other exogenous agents, such as viral or bacterial pathogens signal TORC2 through their cognitive receptors in a host cell? How do metabolites and extracellular nutrients regulate TORC2 activation at the plasma membrane? Could this pathway be explored for the development of new therapies for metabolic disorders and cancer?

Compared with the well characterized lysosome localization of TORC1 activity, the localization of TORC2 is more ambiguous. There could be multiple pools of TORC2, each at a distinct subcellular location. Indeed, TORC2 localization and activity have been observed at several specialized membranous structures, including mitochondria, endosomes, endoplasmic reticulum (ER), and mitochondria-associated ER membrane (MAM), in addition to the plasma membrane (Betz and Hall, 2013). Therefore, it will be important to determine why TORC2 has so many subcellular distributions and what are the main upstream inputs that modulate TORC2 localization. How is TORC2 activation controlled by these inputs? Does one common intracellular input exist that regulates TORC2 activation or does TORC2 sense distinct inputs to enable cell survival in a spatiotemporal manner? It will also be interesting to determine if posttranslational modifications, such as phosphorylation and lipid modifications, of TORC2 components regulate its subcellular targeting to and activation at membranous structures? How are localization and activation of TORC2 coordinated inside the cell?

Dysregulation of TORC2 activity has been linked to many disease processes; this has been firmly established in metabolic diseases and cancer. Efforts are underway to develop selective strategies for targeting TORC2 that are directly involved in disease progression. Because specific and acute small-molecule inhibitors of this kinase are currently unavailable, the identification of TORC2-targeted strategies should be facilitated by the availability of novel mechanisms of TORC2 regulation. One possible approach for TORC2 selective inhibition will be via disruption of complex-specific protein-protein interactions, e.g., the association of mTORC2 with CD146 or other membrane proteins. These targeting approaches would not affect other signaling of TOR pathways or TORC1-dependent negative feedback loops. Alternatively, the rational design of TORC2-specific inhibitors might also be facilitated by the availability of a high resolution 3D structure of TORC2 (Chen et al., 2018; Stuttfeld et al., 2018; Scaiola et al., 2020). The ideal chemical compound might disrupt TORC2 complex assembly, suppress substrate recruitment to the TOR active site, prevent the interaction of upstream regulators, or even inhibit membrane association (e.g., via the PH domain of Sin1). In addition, other novel approaches targeting TORC2 will also hopefully be developed and used in vivo. Indeed, a short, synthetic, single-stranded antisense oligonucleotide (ASO) targeting Rictor specifically has been shown to inhibit mTORC2 activity and reverse the behavioral and neurophysiological abnormalities in PTEN-deficient mice (Chen et al., 2019). Therefore, the development of selective and acute TORC2 inhibitors in future studies might not only provide a firm basis for studying TORC2 signaling but also provide feasible therapeutic strategies for treating TORC2-mediated diseases.

As these ongoing mysteries being revealed and our understanding of these questions becomes increasingly sophisticated, it will not only advance our current view of many vital physiological processes but also provide an important context for therapeutically countering the effects of pathogenic TORC2 in cancer and many other diseases, ultimately improving the quality of human life.

## Author Contributions

JL and YL conceived the concept of article. PA, WX, JL, and YL wrote the manuscript. WX and YL prepared all the figures. All authors contributed to the article and approved the submitted version.

## Conflict of Interest

The authors declare that the research was conducted in the absence of any commercial or financial relationships that could be construed as a potential conflict of interest.

## Publisher’s Note

All claims expressed in this article are solely those of the authors and do not necessarily represent those of their affiliated organizations, or those of the publisher, the editors and the reviewers. Any product that may be evaluated in this article, or claim that may be made by its manufacturer, is not guaranteed or endorsed by the publisher.
